# The relationship between childhood psychological abuse and cyberbullying behavior among graduate students: the mediating role of negative coping style and trait anxiety

**DOI:** 10.3389/fpsyt.2024.1497407

**Published:** 2024-11-29

**Authors:** Yi Shen

**Affiliations:** College of Psychology, Liaoning Normal University, Dalian, China

**Keywords:** graduate student, childhood psychological abuse, negative coping style, trait anxiety, cyberbullying

## Abstract

**Background:**

As a major public health problem, cyberbullying has been received widespread attention in recent years. However, most researches on cyberbullying are mainly focused on adolescents and college students, the underlying mechanisms of cyberbullying among graduate students have been relatively less investigated. From the perspectives of the general aggression model and attachment theory, this study aims to explore the relationship between childhood psychological abuse and cyberbullying behavior among graduate students, as well as the mediating roles of negative coping style and trait anxiety.

**Materials and methods:**

A total of 482 graduate students were surveyed using measures including the Childhood Psychological Abuse Scale, Simplified Coping Style Questionnaire, Self-Rating Anxiety Scale, and Cyberbullying Behavior Scale.

**Results:**

(1) There were significant positive correlations between childhood psychological abuse, negative coping style, trait anxiety, and graduate students’ cyberbullying behavior; (2) Childhood psychological abuse could forecast graduate students’ cyberbullying behavior through the mediating effects of negative coping style and trait anxiety. This mediation process includes two pathways: the independent mediating effect of negative coping style and the chained mediating effect of negative coping style and trait anxiety.

**Conclusion:**

Negative coping style and trait anxiety play mediating roles in the relationship between childhood psychological abuse and cyberbullying behavior among graduate students.

## 1 Introduction

Cyberbullying is a behavior that repeatedly causes harm to individuals or groups with weak self-protection ability through electronic media (1). Cyberbullying victims may exhibit emotional dysregulation, substance abuse, personality disorders, addictive behaviors, and even suicide (2, 3). Cyberbullying behavior is concentrated in the adolescent and adult stages, but the current research on cyberbullying behavior is mainly concentrated on middle school students and college students (4), and research on postgraduates in the early stage of adulthood is relatively less. Therefore, this study mainly examines the current status of cyberbullying behavior among postgraduates and its influencing factors, with the aim of providing theoretical support and empirical evidence for the management departments of universities to formulate effective intervention strategies for cyberbullying behavior among postgraduates.

### 1.1 The relationship between childhood psychological abuse and graduate students’ cyberbullying behavior

According to the General Aggression Model (GAM), cyberbullying behavior is influenced by both environmental factors and individual factors (5). Environmental factors include parenting styles, school education, etc. Individual factors include personality traits, attitudes, motivations, beliefs, values, and other stable psychological characteristics (5, 6). Childhood psychological abuse, as a negative parenting style, refers to a series of inappropriate parenting practices that caregivers continuously repeat during a child’s growth process, including threats, degradation, interference, permissiveness, and neglect (7). According to the attachment theory (8), negative parenting style can lead to anxious attachment, which is related to cyberbullying characteristics (9). Research has found that childhood psychological abuse is positively correlated with hostility towards others in adulthood and can predict cyberbullying behavior (1). Sun et al. (2022) found that perceived childhood psychological abuse can positively predict cyberbullying among college students (10). Based on this, the study proposes Hypothesis 1: Childhood psychological abuse has a positive predictive effect on graduate students’ cyberbullying behavior.

### 1.2 The mediating role of negative coping style and trait anxiety

Negative coping style, as one of the individual factors, is an effective predictor of cyberbullying behavior. Some scholars believe that coping is not a process but a trait, which is a stable response pattern that individuals exhibit in response to stimuli during their interactions with the environment (11). Individuals with more childhood psychological abuse experiences tend to adopt negative coping styles. Yang et al. found that there was a significantly positive relationship between negative coping style and childhood abuse (12). Furthermore, negative coping style increases an individual’s risk of involvement in cyberbullying behavior (13). Therefore, the second hypothesis is proposed:

Hypothesis 2. Negative coping style plays a mediating role in the relationship between childhood psychological abuse and graduate students’ cyberbullying behavior.

Trait anxiety, another important individual factor, is also influenced by childhood psychological abuse. Trait anxiety is a common psychological state with stability and individual differences, mainly manifested as depression, restlessness, tension, fear and other negative emotions, which has a great impact on individuals (14). Studies have found that parents’ excessive protection and interference are not able to effectively guide their children’s emotional problems and may increase their anxiety levels (15). In addition, coping style will affect individuals’ anxiety to varying degrees (16). Coping style are the cognitive and behavioral efforts individuals make to reduce the negative impact on themselves when facing stressors. Previous survey showed that medical graduate students with higher scores on negative coping style have higher average anxiety scores (17). Finally, trait anxiety is closely related to aggressive behavior. Xie et al. found that middle school students’ anxiety and depression were positively correlated with their cyberbullying behavior (18). Therefore, the hypotheses are as follows:

Hypothesis 3. Trait anxiety plays a mediating role in the relationship between childhood psychological abuse and college students’ cyberbullying behavior.

Hypothesis 4. Negative coping style-trait anxiety plays a chain mediating role in the relationship between childhood psychological abuse and college students’ cyberbullying behavior.

## 2 Material and methods

### 2.1 Participants

According to the Bulletin of Education Statistics issued by Liaoning Province, there are 59,000 graduate students in Dalian city and 153,000 graduate students in Liaoning Province. This survey targeted graduate students enrolled at a certain university in Dalian city of Liaoning Province. A total of 550 questionnaires were distributed, with 523 questionnaires returned, and 41 questionnaires excluded as invalid. Ultimately, 482 effective samples were obtained. The number of first-year graduate students is 306, and the number of second-year graduate students is 176. Among them, 270 were male (56%), and 212 were female (44%). There were 401 engineering students (82.32%), 37 science students (7.1%), and 44 liberal arts students (9.1%). 229 graduate students were from urban households (47.51%), and 253 graduate students were from rural households (52.49%). The subjects’ age ranged from 20 to 28 years, with an average age of 23.29 years and a standard deviation of 0.95.

### 2.2 Measures

#### 2.2.1 Childhood psychological abuse scale

The Childhood Psychological Abuse Scale was adopted from Pan et al. (7). The scale consists of five subscales: threatening, neglect, belittling, intermeddling, and indulging, with a total of 23 items. The items were rated on a Likert 5-point scale ranging from “never” (0 score) to “always” (4 score), with higher scores indicating greater levels of psychological abuse experienced by the respondent. In this study, the Cronbach α coefficients for the threatening, neglect, belittling, intermeddling, and indulging subscales were 0.914, 0.863, 0.916, 0.825, and 0.701, respectively.

#### 2.2.2 Simplified coping style questionnaire

The Brief Coping Style Questionnaire was adopted from Xie (19). The scale consists of two subscales: positive coping style and negative coping style, with 12 items in the former and 8 items in the latter, for a total of 20 items. All items were rated on a Likert 4-point scale (1 representing “never” and 4 representing “often”), with higher scores indicating stronger coping style. In this study, the Cronbach α coefficient for the negative coping style subscale was 0.757.

#### 2.2.3 Self-Rating anxiety scale

The Self-Rating Anxiety Scale was adopted from Zung (20), which was revised by Tao and Gao (21). The scale consists of 20 items, rated on a Likert 4-point scale (1 representing “rarely or never” and 4 representing “mostly or all of the time”), with 15 items using negative statements and rated in the order of 1-4, and the remaining 5 items (items 5, 9, 13, 17, and 19) using positive statements and rated in the reverse order of 4-1. Higher scores indicate higher levels of anxiety. The Cronbach α coefficient for the SAS was 0.781.

#### 2.2.4 Cyberbullying behavior scale

The Cyberbullying Behavior Scale was developed by Wright et al. (22). Wang et al. revised the Chinese version of the Cyberbullying Behavior Scale (23). The scale includes 9 items, consisting of 2 subscales: direct cyberbullying behavior (with 5 items) and indirect cyberbullying behavior (with 4 items). The Likert 5-point scale is used, with scores ranging from “never” to “always” being assigned 1 to 5 points respectively. The higher the score, the more cyberbullying behavior. In this study, the Cronbach α coefficients of the direct and indirect cyberbullying behavior subscales were 0.889 and 0.954 respectively.

### 2.3 Procedure

Using a cross-sectional examination, this study adopted a stratified cluster sampling method. The first and second-year graduate students are the main research samples. The researchers randomly selected four joint classes for the test. With the agreement of the research supervisor, the printed questionnaires and informed consent were distributed to graduate students when they are in the class. The project leader read the instructions. The participants volunteered to take the test, then filled in the informed consent form and questionnaires. Only when all the items have been completed, participants can submit the questionnaires.

### 2.4 Data processing

The data were analyzed using SPSS 23.0 for descriptive statistics, partial correlation analysis, reliability analysis, exploratory factor analysis. Mplus 8.1 was used to analysis structural equation modeling. The model fit indices were including the chi-squared goodness-of-fit statistic, the Tucker–Lewis Index (TLI), the comparative fit index (CFI), the root mean square error of approximation (RMSEA), and the standardized root mean square residual (SRMR). TLI and CFI values more than 0.90 indicate a moderate fit, whereas values less than 0.90 signify a poor fit. RMSEA and SRMR values below 0.08 are regarded as a good fit (24). Moreover, Bentler and Chou suggested that a ratio of at least 1:10 between the estimated parameters and the number of samples can ensure the validity of significance tests, and a ratio of at least 1:5 can ensure the reliability of parameter estimates (25).

## 3 Results

### 3.1 Common method bias test

To avoid common method bias in self-reported questionnaire analysis, all questionnaires were anonymous, aiming to enhance the respondents’ truthfulness in answering the items. The exploratory factor analysis was conducted using Harman’s one-factor test on the four questionnaires with 60 items. Without limiting the number of factors, the principal components and the oblique rotation method (Direct-Oblimin) are used to extract common factors. The result showed that KMO = 0.957, *χ²* = 26931.416 (*df* = 1770, *p* < 0.001), which extracted 11 factors with eigenvalues greater than 1 and the first factor explaining 35.528%, below the 40% of judgment standard. Therefore, no common method bias was found in this study.

### 3.2 Descriptive statistics and partial correlation analysis between variables

According to the definition of positive rate of childhood psychological abuse in previous studies (7), the calculation results showed that 72 were positive for childhood psychological abuse (14.94%) and 21 were significantly positive for childhood psychological abuse (4.36%). According to the Chinese norm results, clinical grading of anxiety symptom was calculated according to the boundary value of SAS standard score (21), the results showed that 109 graduate students had mild anxiety (22.61%), 30 graduate students had moderate anxiety (6.25%), and 11 graduate students had severe anxiety (2.28%).

The result about descriptive statistics for variables was in the **Table 1**. After controlling for age and gender, the correlation coefficients between the variables were calculated. The results showed that childhood psychological abuse, negative coping style, trait anxiety, and graduate students’ cyberbullying behavior were significantly positively correlated (see **Table 2**).

**Table 1:** Descriptive statistics for each variable (*N* = 482).

	Minimum	Maximum	*M*	*SD*	Skewness	Kurtosis
childhood psychological abuse	0.000	4.000	0.546	0.628	1.888	5.005
negative coping style	1.000	4.000	2.209	0.587	0.521	0.361
trait anxiety	1.000	3.650	1.854	0.385	1.060	1.729
cyberbullying behavior	1.000	5.000	1.322	0.645	2.681	7.978

**Table 2:** Partial Correlation matrix for each variable (*N* = 482).

	1	2	3	4
1 childhood psychological abuse	1			
2 negative coping style	0.249^***^	1		
3 trait anxiety	0.511^***^	0.392^***^	1	
4 cyberbullying behavior	0.635^***^	0.308^***^	0.514^***^	1

***p < 0.001.

### 3.3 Mediating effect analysis

Since childhood psychological abuse, negative coping style, trait anxiety, and graduate students’ cyberbullying behavior are all latent variables, a structural equation model was established. The non-parametric bootstrap percentile estimation method was used for significance testing with deviation correction, and all variables were standardized. First, a total effect model of childhood psychological abuse predicting graduate students’ cyberbullying behavior was established, and the significance of the total effect coefficient was tested. The results showed that the total effect of childhood psychological abuse on graduate students’ cyberbullying behavior was 0.529 (*p* < 0.001), and the total effect model fit indices were accepted (see **Table 3**).

**Table 3:** Total effect model and mediating model fit indices.

	*χ²*	*df*	CFI	TLI	SRMR	RMSEA
total effect model	60.096	13	0.976	0.961	0.029	0.087
mediating model	176.08	59	0.958	0.944	0.041	0.064

**Figure 1:**
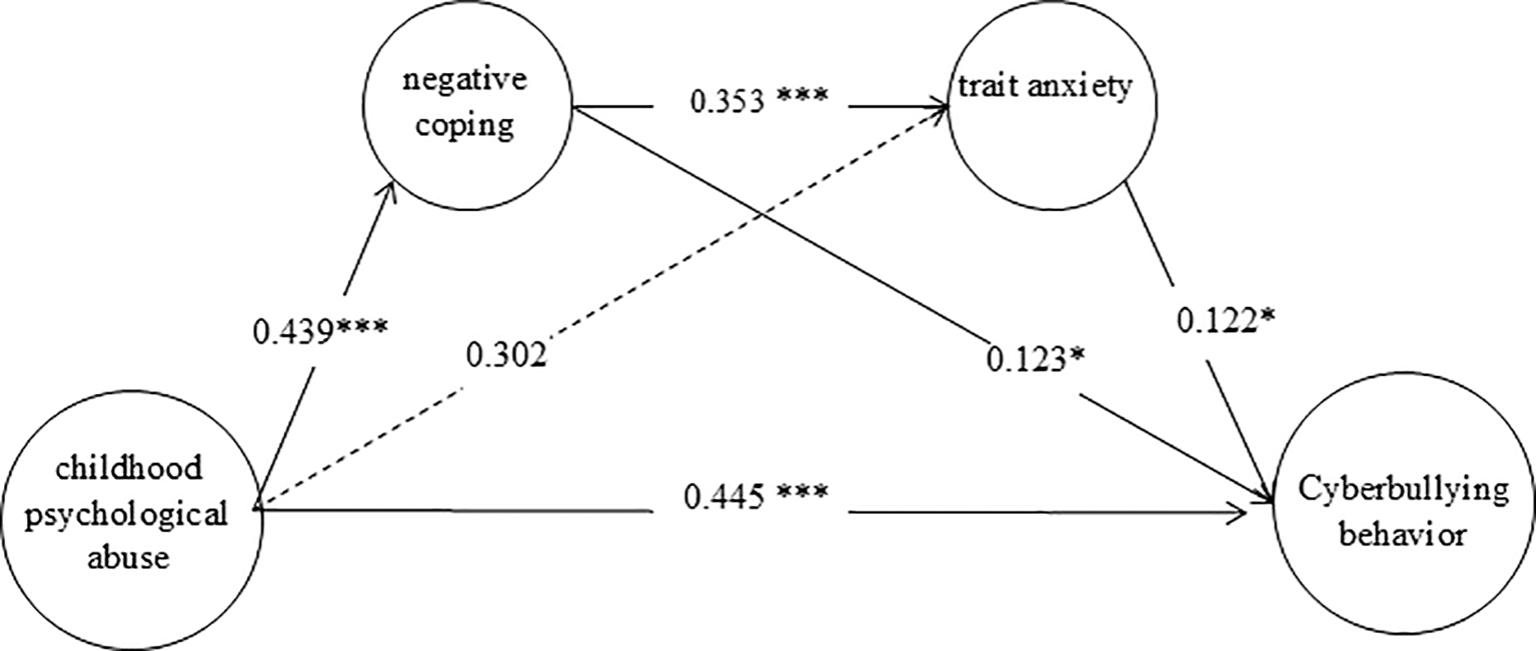
Mediating model of the relationship between childhood psychological maltreatment and graduate students’ cyberbullying behavior.

Secondly, a mediation model was established (see **Figure 1**). The structural equation model analysis showed that the fit indices were good (see **Table 3**), indicating that the model was standard. Among them, the path coefficient from childhood psychological abuse to trait anxiety was not significant (*p* > 0.05). Therefore, negative coping style and trait anxiety play a partial mediating role in the relationship between childhood psychological abuse and graduate students’ cyberbullying behavior, and the mediation role includes two paths: the independent mediating role of negative coping style and the chain mediation role of negative coping style and trait anxiety.

Finally, the 95% confidence interval of the path coefficient was estimated by bootstrapping with 1000 repetitions. The results show that the mediation effect includes two indirect effects. First, the standardized indirect effect 1 generated by the path from childhood psychological abuse → negative coping style → graduate students’ cyberbullying behavior, with a 95% confidence interval of (0.008, 0.117), which does not contain 0, the mediation effect is 0.054, accounting for 10.21% of the total effect. Second, the standardized indirect effect 2 generated by the path from childhood psychological abuse → negative coping style → trait anxiety → graduate students’ cyberbullying behavior, with a 95% confidence interval of (0.003, 0.044), which does not contain 0, the mediation effect is 0.019, accounting for 3.57% of the total effect. Therefore, the total mediation effect is 13.78%.

## 4 Discussion

### 4.1 The relationship between childhood psychological maltreatment and graduate students’ cyberbullying behavior

This study examined the relationship between childhood psychological abuse and graduate students’ cyberbullying behavior by using cross-sectional design. The advantage of this method is that it can find the development similarities and differences of the same age group or different age groups in a short time, and determine the age characteristics of development. Moreover, because several age groups can be investigated and measured at the same time, the information obtained is large, economical and time-consuming. The results showed that childhood psychological abuse had a significant positive predictive effect on graduate students’ cyberbullying behavior, which was basically consistent with the research results of Tian et al. (26). According to the General Aggression Model (GAM), cyberbullying behavior is influenced by interaction of external environmental factors and internal factors (5). Previous studies have shown that traumatic experiences in childhood have a significant impact on an individual’s aggressive behavior in adolescence and adulthood (27). According to the attachment theory (8), individuals experienced psychological abuse in childhood were able to establish an anxious attachment relationship with their parents, which led to the destruction of the “internal working mechanism” for handling various interpersonal relationships and adapting to society when they grew up, thereby causing poor social skills, low empathy, emotional agitation, sensitivity, and suspicion. Meanwhile, when anxiously attached individuals are unable to form and maintain good interpersonal relationships with others, might turn to the online world. When these graduate students used the internet, they would automatically identify some neutral or ambiguous online information as hostile and threatening stimuli, and they would carry out cyberbullying behavior in order to protect themselves from being harmed. In addition, graduate student who experienced more childhood psychological abuse would take more negative defensive strategies in interpersonal communication (28). They would make incorrect attributions about others’ intentions, thereby producing hostile attribution bias (29), and thus choosing to carry out relatively covert cyberbullying behavior.

From a cultural perspective, cyberbullying behavior is closely related to cultural background. Previous research found that there was no significant difference in traditional bullying behaviors between the two samples. However, Canadian adolescents were more likely to engage in cyberbullying than Chinese adolescents (30). In the Chinese culture that values peace, people try to avoid conflict. In Western cultures, which are based on innate human rights, it may be easier to fight against threatening stimuli. However, there is a lack of research on cross-cultural comparison of cyberbullying, and the harm of cyberbullying has obviously existed, so it is very necessary to study the occurrence, development, prevention and intervention measures of cyberbullying in different cultural backgrounds.

### 4.2 The mediation effects of negative coping style and trait anxiety

This study found that negative coping style mediates the relationship between childhood psychological abuse and graduate students’ cyberbullying behavior, verifying the research hypothesis. Previous study found that the development of negative coping strategies was affected by the environment, especially the impact of maltreatment (31). When parents psychologically abuse children, children will evaluate the situation as threatening or hostile information (32). If situational cues are evaluated as threatening or hostile information, individuals will adopt corresponding coping style. Because children are in a vulnerable position in the family under Chinese culture, they are more likely to develop negative coping style. Yang et al. also found that childhood maltreatment and neglect can positively predict adult negative coping style (12). Thus, coping style is the result of the interactive effects of an individual’s stable factors and the environment, and is an important indicator of social adaptation and development. When individuals encounter stressful events, they will adopt habitual coping styles to manage and regulate the demands arising from the stressful events. Since graduate students who experienced psychological abuse in childhood have sensitive and suspicious characteristics, they have a lower threshold for evaluating the danger of some information in the online environment than normal people. When they pay attention to the information, they will subsequently activate physiological arousal, automatically encoding the information as hostile and threatening stimuli, and subsequently activating their negative coping style, thereby taking cyberbullying behaviors.

This study also found that negative coping style and trait anxiety mediate the relationship between childhood psychological abuse and graduate students’ cyberbullying behavior, validating the research hypothesis. Yang et al. found that childhood abuse and neglect were positively related to the negative coping (12). When children encounter parental psychological abuse, using negative coping style cannot effectively solve the problems they face, so they often experience anxiety, which leads to trait anxiety. Trait anxiety is a common emotional disorder that is related to a lack of emotional regulation ability and the reinforcement of negative emotions. Research has found that trait anxiety is positively correlated with cyberbullying behavior, i.e., the higher the level of trait anxiety, the more frequent the cyberbullying behavior (33). Graduate students with childhood psychological abuse experiences are more likely to engage in attacking behavior through more covert means of the internet to alleviate their anxiety. Therefore, the research findings validate the research hypothesis.

### 4.3 Limitations and future directions

Despite the important findings of this study, there are some limitations that need to be addressed. First, the present study employed a self-report questionnaire. Although common method bias was not found in this study, future studies could use other methods, such as peer-rating or experimental design, to enhance the validity of the study results. Especially, diverse data collection methods with a mix of quantitative and qualitative sources would be used to alleviate common method biases. Second, the sample of the present study was recruited from a polytechnic university, limiting the generalizability of the study findings. Future studies could recruit a more diverse sample of participants from other disciplines or universities. Third, the present study employed a cross-sectional research design, which can only examine correlations between variables, not causality. Future studies could combine longitudinal research to examine the causality between them. Finally, this study only examined negative coping style and trait anxiety as mediating variables. The additional mediating variables would be considered to incorporate into the model, such as personality trait, attachment style, et al.

## 5 Conclusions

This study found that childhood psychological abuse could forecast graduate students’ cyberbullying behavior through the mediating effects of negative coping style and trait anxiety. This mediation process includes two pathways: the independent mediating effect of negative coping style and the chained mediating effect of negative coping style and trait anxiety. Thus, childhood psychological abuse may have long-term effects on the development of graduate students’ cyberbullying behavior, in which negative coping style and trait anxiety play an important mediating role. Therefore, in the prevention and intervention of graduate students’ cyberbullying behavior, education department should pay attention to the long-term effects of parenting style on individual psychological development, and help individuals form positive coping style and alleviate trait anxiety, thereby reducing their risk of engaging in cyberbullying behavior.

## Data Availability

The raw data supporting the conclusions of this article will be made available by the authors, without undue reservation.
